# Identification of patients undergoing chronic kidney replacement therapy in primary and secondary care data: validation study based on OpenSAFELY and UK Renal Registry

**DOI:** 10.1136/bmjmed-2023-000807

**Published:** 2024-04-18

**Authors:** Shalini Santhakumaran, Louis Fisher, Bang Zheng, Viyaasan Mahalingasivam, Lucy Plumb, Edward PK Parker, Retha Steenkamp, Caroline Morton, Amir Mehrkar, Sebastian Bacon, Sue Lyon, Rob Konstant-Hambling, Ben Goldacre, Brian MacKenna, Laurie A Tomlinson, Dorothea Nitsch

**Affiliations:** 1 UK Renal Registry, UK Kidney Association, Bristol, UK; 2 Bennett Institute for Applied Data Science, University of Oxford, Oxford, UK; 3 London School of Hygiene and Tropical Medicine, London, UK; 4 Population Health Science Institute, University of Bristol, Bristol, UK; 5 UKKA Patient Council, UK Kidney Association, Bristol, UK; 6 Specialised Commissioning, NHS England, Cheshire, UK

**Keywords:** COVID-19, kidney failure, chronic, dialysis, kidney transplantation, epidemiology

## Abstract

**Objective:**

To validate primary and secondary care codes in electronic health records to identify people receiving chronic kidney replacement therapy based on gold standard registry data.

**Design:**

Validation study using data from OpenSAFELY and the UK Renal Registry, with the approval of NHS England.

**Setting:**

Primary and secondary care electronic health records from people registered at 45% of general practices in England on 1 January 2020, linked to data from the UK Renal Registry (UKRR) within the OpenSAFELY-TPP platform, part of the NHS England OpenSAFELY covid-19 service.

**Participants:**

38 745 prevalent patients (recorded as receiving kidney replacement therapy on 1 January 2020 in UKRR data, or primary or secondary care data) and 10 730 incident patients (starting kidney replacement therapy during 2020), from a population of 19 million people alive and registered with a general practice in England on 1 January 2020.

**Main outcome measures:**

Sensitivity and positive predictive values of primary and secondary care code lists for identifying prevalent and incident kidney replacement therapy cohorts compared with the gold standard UKRR data on chronic kidney replacement therapy. Agreement across the data sources overall, and by treatment modality (transplantation or dialysis) and personal characteristics.

**Results:**

Primary and secondary care code lists were sensitive for identifying the UKRR prevalent cohort (91.2% (95% confidence interval (CI) 90.8% to 91.6%) and 92.0% (91.6% to 92.4%), respectively), but not the incident cohort (52.3% (50.3% to 54.3%) and 67.9% (66.1% to 69.7%)). Positive predictive values were low (77.7% (77.2% to 78.2%) for primary care data and 64.7% (64.1% to 65.3%) for secondary care data), particularly for chronic dialysis (53.7% (52.9% to 54.5%) for primary care data and 49.1% (48.0% to 50.2%) for secondary care data). Sensitivity decreased with age and index of multiple deprivation in primary care data, but the opposite was true in secondary care data. Agreement was lower in children, with 30% (295/980) featuring in all three datasets. Half (1165/2315) of the incident patients receiving dialysis in UKRR data had a kidney replacement therapy code in the primary care data within three months of the start date of the kidney replacement therapy. No codes existed whose exclusion would substantially improve the positive predictive value without a decrease in sensitivity.

**Conclusions:**

Codes used in primary and secondary care data failed to identify a small proportion of prevalent patients receiving kidney replacement therapy. Codes also identified many patients who were not recipients of chronic kidney replacement therapy in UKRR data, particularly dialysis codes. Linkage with UKRR kidney replacement therapy data facilitated more accurate identification of incident and prevalent kidney replacement therapy cohorts for research into this vulnerable population. Poor coding has implications for any patient care (including eligibility for vaccination, resourcing, and health policy responses in future pandemics) that relies on accurate reporting of kidney replacement therapy in primary and secondary care data.

**WHAT IS ALREADY KNOWN ON THIS TOPIC**

- The OpenSAFELY platform linked multiple sources of data from electronic health records for research into covid-19 and found that people receiving kidney replacement therapy had a higher risk of death from covid-19
- For primary care data, accurate coding is needed to ensure access to timely vaccination and antiviral treatment
- Accurately identifying existing and new patients receiving kidney replacement therapy with coded electronic health records is essential for research and resourcing in this vulnerable population

**WHAT THIS STUDY ADDS**

- Cohorts receiving kidney replacement therapy, particularly patients starting chronic dialysis, could not be accurately identified in primary and secondary care electronic health records from standard kidney replacement therapy codes only
- About half of the people identified by dialysis codes did not meet the gold standard definition for chronic dialysis of the UK Renal Registry (UKRR), implying that analyses that do not use UKRR data cannot reliably distinguish between people who have had acute dialysis from those receiving chronic kidney replacement therapy
- No primary or secondary care codes existed whose exclusion could substantially improve accuracy

**HOW THIS STUDY MIGHT AFFECT RESEARCH, PRACTICE, OR POLICY**

- Linking to registry data when using data from electronic health records to identify people receiving chronic kidney replacement therapy is valuable
- Patients starting kidney replacement therapy who become eligible for targeted treatments, such as vaccination or health policy responses (eg, shielding in the context of future pandemics) might not be identified in a timely way by primary and secondary care codes
- Inaccuracies and variability in coding can affect subsequent resourcing levels of kidney care and its equitability across the population

## Introduction

Chronic kidney disease is a major risk factor for death from covid-19 disease.^1^ The most severe stage of chronic kidney disease is when people require kidney replacement therapy (dialysis or kidney transplantation) to stay alive. Analysis of primary care data held in the OpenSAFELY platform showed that people coded as recipients of kidney replacement therapy, after adjustment for other comorbid conditions, had at least a threefold increased risk of death related to covid-19, exceeding that of other high risk groups.^2^

Patients with chronic kidney disease can be identified in electronic health records by coded activity from primary care, but not all people with chronic kidney disease are coded by their doctor, even though they have laboratory evidence indicating chronic kidney disease.^3^ A subgroup of patients with advanced chronic kidney disease (stages 4-5) might be exclusively managed in secondary care, and corresponding laboratory values could be missing from their primary care record^4^. Also, when patients start kidney replacement therapy, a delay in informing their doctor can occur. These discrepancies result in potentially inaccurate identification of these patients from their primary care record. Similar concerns exist for secondary care electronic health records; although kidney replacement therapy will only start in a secondary care setting, accuracy of coding of routine data is a concern,^5–7^ but few published data exist on the quality of coding of kidney replacement therapy.^8 9^ Previous work linking data from the UK Renal Registry (UKRR) with Hospital Episode Statistics (NHS secondary care data) showed that using only Hospital Episode Statistics to identify ongoing chronic dialysis was satisfactory in only a subgroup of English centres, because not all centres reported dialysis consistently.^9^

OpenSAFELY is a secure, health analytics platform, set up to monitor the effect of covid-19 on health outcomes. OpenSAFELY contains electronic health records from general practices covering 40% of the population of England.^10^ Because of the risks related to covid-19 disease, correctly identifying people receiving kidney replacement therapy when analysing data on vaccination^11^ and antiviral treatment is important.^12^ Therefore, a linkage was set up with the UKRR that collects data on patients treated with kidney replacement therapy from all renal centres in the UK. With this unique linked data resource, we compared coding of kidney replacement therapy in primary and secondary care data with UKRR data, to understand whether patients have been accurately identified in existing analyses of linked primary and secondary care data.

## Methods

### Study design

With the approval of NHS England, we conducted a validation study based on data from primary care electronic health records from general practices in England, supplied by the electronic health records vendor the Phoenix Partnership (TPP), as well as linked secondary care data from the NHS Digital Secondary Uses Services and data from UKRR.

### Data sources

All data were linked, stored, and analysed securely within the OpenSAFELY platform,^10^ as part of the NHS England OpenSAFELY covid-19 service. The OpenSAFELY-TPP dataset is based on primary care data from about 45% of general practices in England that use TPP software. Data are pseudonymised and include coded diagnoses, drug treatments, and physiological parameters. No free text data are included. Primary care data managed by the general practices software provider TPP are linked to other pseudonymised datasets through OpenSAFELY. In this study, we included secondary care data from the Admitted Patient Care Statistics and Outpatient Attendances datasets from NHS Digital, and gold standard data on recipients of kidney replacement therapy from UKRR. All renal centres in the UK submit data to the UKRR on all recipients of kidney replacement therapy, derived from the centre's renal IT system. Data are validated and cleaned to produce a finalised database annually. A wide range of clinical and personal characteristics are collected by the UKRR, but data linked within OpenSAFELY were limited to a cohort identifier, treatment modality, and kidney care centre. All code is shared openly for review and re-use under an MIT (Massachusetts Institute of Technology) open license.^13^ Detailed pseudonymised patient data are potentially re-identifiable and therefore not shared.

### Study population

We included all patients registered with an OpenSAFELY-TPP practice on 1 January 2020. Children (defined as age <18 years) were analysed separately. Age, sex, region of residence, index of multiple deprivation group (divided by quintiles), diabetes, and hypertension were determined from the primary care records as of 1 January 2020. Data for sex were taken from information in electronic health records rather than from patient reported gender. Ethnic group was determined from the latest available information in primary and secondary care data.

### Definition of kidney replacement therapy status

We compared prevalent and incident cohorts receiving kidney replacement therapy, identified in the primary care, secondary care, and UKRR datasets. The main analyses presented are for a prevalent cohort of people receiving kidney replacement therapy on 1 January 2020 and an incident cohort of people starting kidney replacement therapy for the first time during 2020. Dialysis and transplant cohorts were considered separately, as well as a cohort for any kidney replacement therapy. We also examined prevalent cohorts as of 1 January 2021 and 1 January 2022. We report specific definitions for each data source.

For UKRR data, prevalent patients were defined as people receiving kidney replacement therapy on 1 January 2020. Incident patients were those who started kidney replacement therapy (dialysis or pre-emptive transplant) in 2020. Patients returning to dialysis after a failed transplant were not considered incident, or those who recovered kidney function within 90 days of starting treatment. Patients who received dialysis for acute kidney injury were only included if their dialysis was subsequently recoded by the renal centre as being for end stage kidney disease, or if the patient was still receiving kidney replacement therapy at 90 days and not confirmed by the renal centre as still receiving acute dialysis.

In the primary and secondary care data, patients in the prevalent cohort had a recorded code indicating kidney replacement therapy on or before 1 January 2020. The treatment modality was based on the latest recorded code. Some kidney replacement therapy codes do not specify modality; also, patients could have dialysis and transplant codes recorded on the same day. In these cases, patients were included in the any kidney replacement therapy definition but not under a specific modality. The incident cohort was defined as people whose first instance of a recorded code indicating kidney replacement therapy was during 2020. The coding systems used were CTV3 (NHS Read codes used in primary care), OPCS-4 (UK codes for operations, procedures, and interventions in UK secondary care), and ICD-10 (international classification of diseases, 10th revision, international diagnosis codes in secondary inpatient care). The code lists (online supplemental file 1) are published on the OpenCodelists website.^14^ The primary care code lists are those used in previous OpenSAFELY studies, whereas the secondary care code lists were developed for this study.

10.1136/bmjmed-2023-000807.supp1 Supplementary data

The UKRR prevalent cohort did not include people who received kidney replacement therapy in the past but had recovered or stopped treatment by 1 January 2020. This group was included in the primary and secondary care cohorts under the definition described above. We considered that patients who had some recovery of renal function and no longer required kidney replacement therapy might have had an earlier stage chronic kidney disease code superseding their last kidney replacement therapy code. Therefore, in a sensitivity analysis, we excluded people from the primary and secondary care prevalent cohorts who had a chronic kidney disease code (SNOMED-CT (Systematised Nomenclature of Medicine Clinical Terms) for primary care and ICD-10 for secondary care, online supplemental file 1) which was more recent than their last kidney replacement therapy code.

### Data analysis

We identified incident and prevalent cohorts receiving kidney replacement therapy based on the primary care, secondary care, and UKRR definitions. Personal characteristics were described for each cohort, and compared with those for all people in England receiving kidney replacement therapy on 1 January 2020, as reported by the UKRR. The UKRR is considered the gold standard, and therefore the sensitivity (percentage of true positive results detected) and positive predictive value (percentage of positive values that are true positive values) of the primary and secondary care definitions were calculated. Agreement across the three data sources was illustrated with Euler diagrams. Measures of accuracy and agreement were calculated by personal characteristics (age, sex, ethnic group, region of residence, and index of multiple deprivation group) and by risk factors for chronic kidney disease (presence of diabetes and hypertension).

For people in the UKRR cohorts who were not identified in primary or secondary care data, we examined whether these patients had any chronic kidney disease codes. For patients identified in primary and secondary care who were not in the UKRR cohorts, we checked which codes were being applied. For secondary care, as an indication of acute dialysis, we reported how many patients had at least one day of critical care in the same hospital admission. For primary care, we used the estimated glomerular filtration rate grouped by stage of chronic kidney disease^15^ (taking the most recent measurement before the prevalent date) to describe the level of kidney function in these patients.

Measures of accuracy and agreement were repeated for dialysis and transplant treatment modalities, and discrepant modalities were reported. For the incident cohort, we compared the year of the start of kidney replacement therapy across the three data sources and looked at primary care codes recorded within three months of starting kidney replacement therapy. Corresponding analysis of prevalent cohorts as of 1 January 2021 and 2022 were reported separately. For 2022, the UKRR cohort was based on provisional data submitted by kidney care centres as part of the covid-19 data collection, which has not undergone full validation and cleaning. All numbers <7 were redacted and the remaining numbers rounded to the nearest five, in accordance with OpenSAFELY guidance. Totals were calculated after rounding and so might not be consistent across tables.

### Software and reproducibility

Data management was performed with Python 3.8, with analysis carried out with R.^16^ Code for data management and analysis as well as code lists are archived online.^13^

### Patient and public involvement

No patients were recruited as part of the study. SL is chair of the UK Kidney Association (UKKA) Patient Council, who are actively involved in the work of the UKRR. Findings of research based on UKRR data are regularly disseminated to patients and the public by the UKKA. OpenSAFELY has developed a publicly available website https://opensafely.org/ through which they invite any patient or member of the public to make contact regarding the broader OpenSAFELY project.

## Results

The study population was 19 445 260 people; 38 745 unique people registered with an OpenSAFELY-TPP practice were recorded as receiving kidney replacement therapy at the start of 2020 in one of the three data sources. Comparing UKRR cohorts in the OpenSAFELY-TPP database with data from all English renal centres (online supplemental table S2), we found that a higher proportion of the linked cohort were in the white ethnic group (80% *v* 72% throughout England). Differences in regional representation of patients receiving kidney replacement therapy relative to the total population receiving kidney replacement therapy in UKRR were in line with TPP coverage.^17^ In particular, a smaller proportion of the linked cohort were from London (8% *v* 26% throughout England) because the number of general practices in London that use TPP software is small. The prevalence of kidney replacement therapy in the population was similar in the whole of England (0.13%) and the OpenSAFELY subset (0.12%).

### Comparison of prevalent cohorts

**Figure 1:**
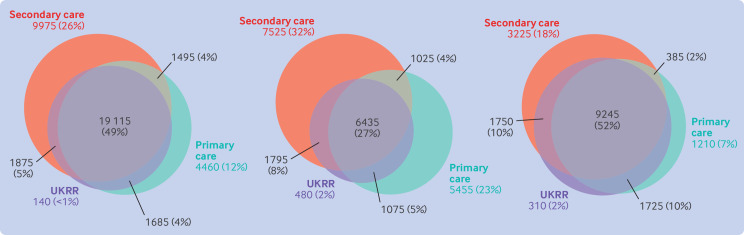
Euler diagrams showing agreement for prevalent adult patients (recorded as receiving kidney replacement therapy on 1 January 2020 in UK Renal Registry (UKRR) data, or primary or secondary care data) for all kidney replacement therapy (left), for those receiving dialysis (middle), and for those who had a kidney transplant (right). All numbers rounded to nearest 5

**Figure 2:**
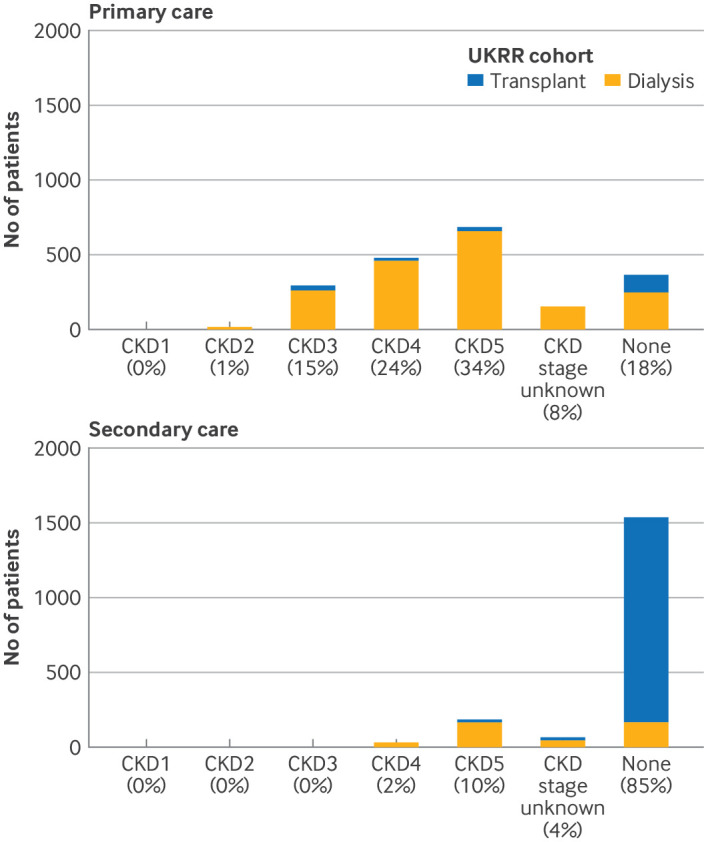
Latest chronic kidney disease (CKD) status, based on CKD stage, in primary and secondary care data for adults in the prevalent UK Renal Registry (UKRR) cohort on 1 January 2020 with no kidney replacement therapy codes in primary care data (top) and secondary care data (bottom). All numbers rounded to nearest 5

Of the 38 745 people receiving kidney replacement therapy at the start of 2020 in any of the three data sources, 49% were in all three cohorts (figure 1). For people who were in both the UKRR and primary care cohorts, 89% had the same treatment modality recorded (rising to 92% for secondary care). The agreement was lower for dialysis (27% in all data sources, figure 1) than for kidney transplantation (52%, figure 1). A small number of people (≤2% for any treatment modality) were identified only in UKRR data. Looking at the primary and secondary care data only, however, more of the UKRR cohort were missed (9% and 8%, respectively). People missed by primary care codes were mostly patients receiving dialysis and most had a chronic kidney disease code in the primary care data (figure 2). In contrast, people missed in the secondary care data were mostly patients who had received a kidney transplant, and not many had a chronic kidney disease code in secondary care (figure 2). Patients who received dialysis recorded only in primary care data numbered 5415 people (23% of the total) compared with 7505 (32%) only in secondary care data, with a further 990 (4%) patients in both primary and secondary care data but not in UKRR data. Some of this discrepancy is because of differences in treatment modality: 1700 people in the primary care dialysis cohort were recorded as having received a transplant in UKRR data.

Patient age was similar in all cohorts, and the UKRR cohort had a slightly higher proportion of men (online supplemental table S3). The distribution of ethnic groups was similar among the prevalent transplant cohorts, but for dialysis, a larger percentage of patients in the primary care (81%) and secondary care (83%) cohorts were in the white ethnic group compared with patients in the UKRR cohort (77%). Online supplemental table S4 shows agreement by personal characteristics for prevalent patients receiving kidney replacement therapy in the three cohorts.

#### Sensitivity and positive predictive value

Table 1 shows the sensitivity and positive predictive values of the primary and secondary care definitions for all kidney replacement therapy, with the UKRR cohort as the gold standard, overall and by personal characteristics (online supplemental table S5 shows the corresponding results by treatment modality). Overall, sensitivity was high (>91%) for both primary and secondary care codes for identifying the prevalent UKRR kidney replacement therapy cohort, but poorer for dialysis only (77%, 95% confidence interval (CI) 76% to 78% for primary care and 84%, 83% to 85% for secondary care) and to a lesser extent for kidney transplant. The positive predictive value was lower than the sensitivity value (78%, 78% to 78% for primary care and 65%, 64% to 66% for secondary care), particularly for dialysis (54%, 53% to 55% for primary care and 49%, 48% to 50% for secondary care), and was better for kidney transplant (87%, 86% to 88% for primary care and 75%, 74% to 76% for secondary care). If both primary and secondary care definitions were met, we saw an increase in the positive predictive value to 93% (92% to 93%), accompanied by a decrease in sensitivity to 84% (83% to 84%). A broader definition of a primary or secondary care code increased sensitivity to 99% (99% to 99%) but the positive predictive value decreased to 59% (58% to 59%).

**Table 1:** Sensitivity and positive predictive value of primary and secondary care definitions (with UK Renal Registry cohort as gold standard) by personal characteristics, for prevalent kidney replacement therapy cohorts at the start of 2020

Category	Primary care		Secondary care	
	Sensitivity (%) (95% CI)	Positive predictive value (%) (95% CI)	Sensitivity (%) (95% CI)	Positive predictive value (%) (95% CI)
All patients	91 (91 to 92)	78 (78 to 78)	92 (92 to 92)	65 (64 to 65)
Age (years)				
18-39	95 (94 to 96)	77 (76 to 78)	91 (90 to 92)	67 (65 to 69)
40-49	94 (93 to 95)	81 (80 to 82)	89 (88 to 90)	68 (66 to 70)
50-59	93 (92 to 94)	83 (82 to 84)	91 (90 to 92)	71 (70 to 72)
60-69	91 (90 to 92)	80 (79 to 81)	92 (91 to 93)	65 (64 to 66)
70-79	87 (86 to 88)	74 (73 to 75)	95 (94 to 96)	59 (57 to 61)
≥80	82 (80 to 84)	63 (61 to 65)	94 (93 to 95)	56 (54 to 58)
Sex				
Women	92 (91 to 93)	74 (73 to 75)	93 (92 to 94)	63 (62 to 64)
Men	91 (91 to 91)	80 (79 to 81)	91 (91 to 91)	66 (65 to 67)
Ethnic group				
White	91 (91 to 91)	77 (76 to 78)	92 (92 to 92)	62 (61 to 63)
Mixed	94 (91 to 97)	79 (75 to 83)	96 (94 to 98)	69 (63 to 75)
South Asian	90 (89 to 91)	86 (85 to 87)	95 (94 to 96)	77 (75 to 79)
Black	90 (88 to 92)	86 (84 to 88)	92 (90 to 94)	77 (74 to 80)
Other	92 (89 to 95)	83 (79 to 87)	89 (86 to 92)	74 (69 to 79)
Index of multiple deprivation group				
1 (most deprived)	89 (88 to 90)	78 (77 to 79)	94 (93 to 95)	66 (65 to 67)
2	90 (89 to 91)	79 (78 to 80)	93 (92 to 94)	66 (65 to 67)
3	92 (91 to 93)	78 (77 to 79)	92 (91 to 93)	63 (62 to 64)
4	93 (92 to 94)	76 (75 to 77)	91 (90 to 92)	63 (61 to 65)
5 (least deprived)	93 (92 to 94)	77 (76 to 78)	90 (89 to 91)	64 (62 to 66)

CI, confidence interval.

**Figure 3:**
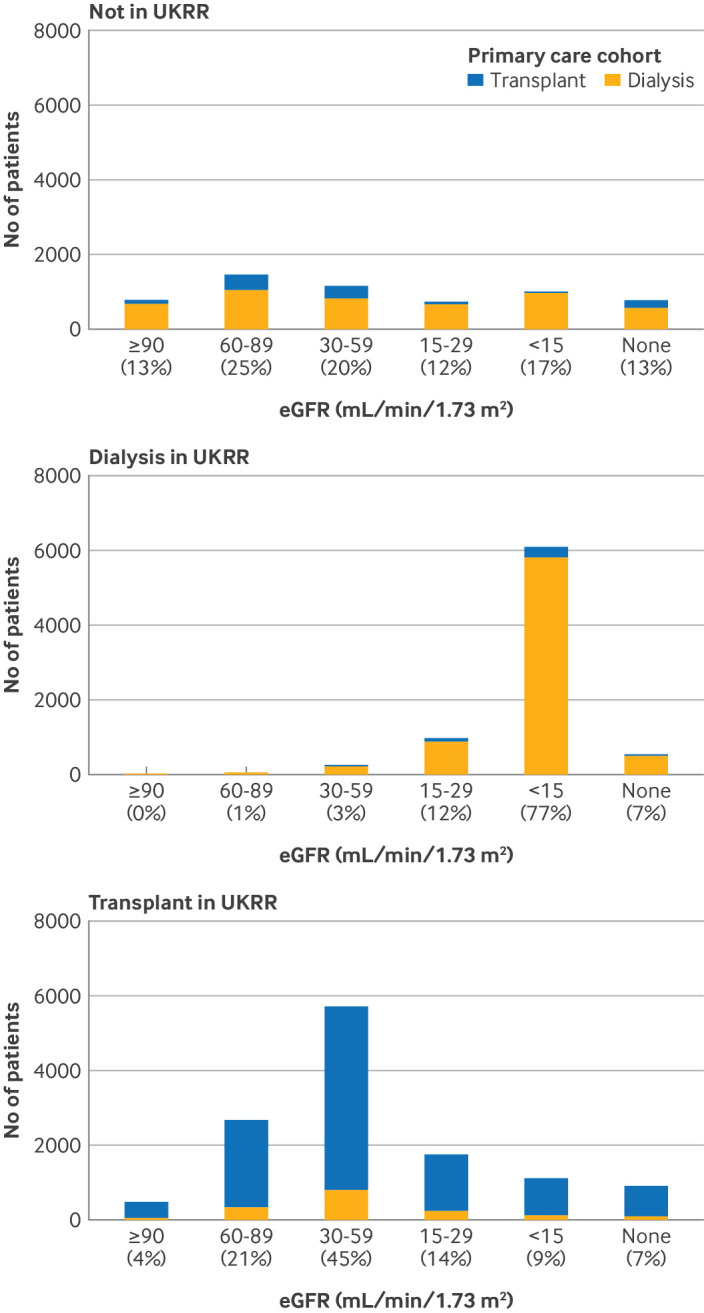
Most recent estimated glomerular filtration rate (eGFR) measurement (before prevalent date) in primary care data, for adults in the prevalent primary care cohort on 1 January 2020, by treatment modality (transplant or dialysis), recorded in UK Renal Registry (UKRR) on 1 January 2020. All numbers rounded to nearest 5; n≤7 and kidney replacement therapy recipients with unknown modality in primary care data (<1% of total) are not shown

For primary care data, excluding patients who might have recovered some kidney function, as indicated by a chronic kidney disease code after their most recent kidney replacement therapy code, increased the positive predictive value (79%, 95% CI 79% to 80%) but reduced sensitivity (75%, 74% to 75%). For the prevalent dialysis cohort, we saw a higher positive predictive value of 84% (83% to 85%) in the primary care data when the analysis was restricted to people with a latest estimated glomerular filtration rate (before the prevalent date) of <15 mL/min/1.73 m^2^. This restriction also led to a reduction in sensitivity to 78% (77% to 79%); furthermore only 76% of the UKRR dialysis cohort had a latest estimated glomerular filtration rate of <15 mL/min/1.73 m^2^ in the primary care data. Figure 3 shows the distribution of measurements of estimated glomerular filtration rate by treatment modality.

Sensitivity of the kidney replacement therapy codes decreased with age for primary care data but the opposite was true for secondary care data. For dialysis only, sensitivity and positive predictive values were higher in the older age groups in both settings. In general, sensitivity was slightly higher for women than for men, but the positive predictive value was lower. Positive predictive values were lower for white patients than for black and South Asian patients in both primary and secondary care data, for overall kidney replacement therapy and dialysis cohorts, with no clear differences in sensitivity. Sensitivity decreased with increasing area level deprivation for primary care data but increased for secondary care data.

#### Recipients of kidney replacements in primary and secondary care cohort but not in UKRR cohort

Both primary and secondary care definitions identified people who did not receive chronic kidney replacement therapy according to UKRR data, and most were coded by primary and secondary care data as receiving dialysis. Of the 5955 people coded as receiving kidney replacement therapy in primary care data who were not in UKRR data, 81% were coded as receiving dialysis and 19% as receiving a kidney transplant. An estimated glomerular filtration rate measurement was available for 87% of these patients, who had higher values than patients who were also identified as recipients of kidney replacement therapy in UKRR data, particularly for dialysis (figure 3).

Among the 11 470 people identified by kidney replacement therapy codes in secondary care who were not in the UKRR cohort, 74% had received dialysis and 26% had kidney transplant related codes. Online supplemental figure S1 shows the most common codes for these patients (recorded for at least 5%), compared with the frequency of these codes for people in the UKRR cohort. In the primary care data, codes for acquired arteriovenous fistula and transplant nephrectomy were applied more frequently to people who were not recipients of kidney replacement therapy in UKRR data compared with those who were. Among the secondary care cohort, the code for dialysis not elsewhere classified was applied to 6540 (57%) patients who were not part of the UKRR cohort, and 4885 of these had some critical care during the same episode. This code was only applied to 19% of patients who were also recipients of kidney replacement therapy in UKRR data, and only 12% of those received any critical care during their stay.

#### Agreement in children

For all kidney replacement therapy, agreement was lower in children (30% in all three data sources, online supplemental figure S2A) than for adults, and with a larger discrepancy between dialysis (7%, online supplemental figure S2B) and transplant (63%, online supplemental figure S2C) than was seen for adults. Poor agreement for the dialysis cohorts was because of inclusion of patients in the primary and secondary care definitions who were not part of the UKRR cohorts, and not many UKRR patients were missed. Analysis of primary and secondary care codes was not performed because of small numbers.

#### Agreement in subsequent years

Online supplemental figure S3 shows the Euler diagrams for agreement for prevalent kidney replacement therapy cohorts on 1 January 2021 and 2022. Sensitivity and positive predictive values were similar for the primary care definition (sensitivity 91%, 95% CI 91% to 91% for 2021 and 90%, 90% to 91% for 2022; positive predictive values 77%, 76% to 77% for 2021 and 2022). Sensitivity of the primary care definition increased slightly (94%, 93% to 94% for 2021 and 95%, 94% to 95% for 2022) but we saw a marked decrease in the positive predictive value (60%, 59% to 61% for 2021 and 57%, 57% to 58% for 2022) because of a large increase in the numbers identified by the secondary care definition.

### Comparison of incident cohorts

**Figure 4:**
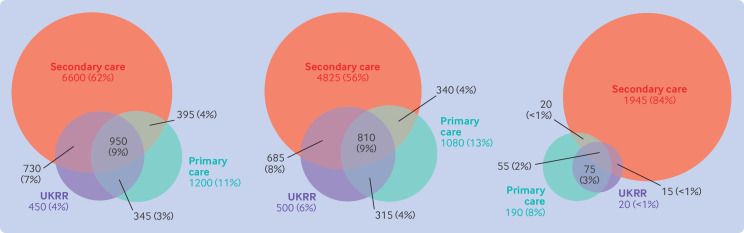
Euler diagrams showing agreement for incident adult patients (starting kidney replacement therapy during 2020 in UK Renal Registry (UKRR) data, or primary or secondary care data) for all kidney replacement therapy (left), for those receiving dialysis (middle), and for those who had a kidney transplant (right). All numbers rounded to the nearest 5

In 2020, the number of people starting kidney replacement therapy (who were registered in an OpenSAFELY-TPP practice) was 2475 based on UKRR data, 2880 in the primary care data, and 8730 in the secondary care data. Less than 10% of the people identified as 2020 incident patients in any of the data sources were found in all three sources, with most people identified in only secondary care data (figure 4). Sensitivity and positive predictive values of primary and secondary care data compared with the gold standard UKRR data were low throughout (online supplemental table S6). Some of the UKRR 2020 incident kidney replacement therapy cohort that were not part of the 2020 incident cohorts in primary or secondary care were identified in corresponding 2019 or 2021 cohorts (about a third for primary care and more than two thirds for secondary care). We found that 670 patients in UKRR data had no record of kidney replacement therapy in primary care data; almost all were patients receiving dialysis in UKRR data and 75% had a chronic kidney disease code. Online supplemental figure S1 shows the most common codes for people in the primary and secondary care incident cohorts and not in the UKRR cohort, compared with the frequency of these codes for people in the UKRR incident cohort.

Of the 2020 incident patients receiving dialysis in UKRR data, analysis of their primary care data showed that 50% had no kidney replacement therapy code within three months before or after the UKRR start date, 13% had a code for haemodialysis, 8% compensation for renal failure, 6% peritoneal dialysis, and 6% end stage renal failure. All other codes were applied to <5% of patients. Less than 5% of UKRR 2020 incident (pre-emptive) patients who had received a transplant had no primary care kidney replacement therapy code within three months of the UKRR start date; 53% had renal transplant, 19% had live donor renal transplant, and 6% had transplant kidney codes.

## Discussion

### Main findings

We used the OpenSAFELY platform to compare how electronic health records in primary and secondary care in England identified cohorts of people receiving kidney replacement therapy. We found that when used separately, primary and secondary care electronic health records had high sensitivity but low positive predictive value when identifying people currently receiving kidney replacement therapy, with accuracy higher for identifying kidney transplantation than dialysis. We found some variation in accuracy by personal characteristics (in particular, lower accuracy in children), and different patterns were seen in primary and secondary care data. When patients in UKRR data were identified in primary or secondary care data, agreement for treatment modality with UKRR data was high. Prevalent patients in UKRR data who were not coded as receiving kidney replacement therapy in primary care data for the most part had a diagnosis of chronic kidney disease. This group was more likely to be recipients of dialysis in UKRR data. In contrast, most patients in UKRR data with no kidney replacement therapy code in secondary care data also had no chronic kidney disease code, and were largely recipients of kidney transplants in UKRR data. Start dates for kidney replacement therapy were inaccurate in primary care, with half of the UKRR incident cohort having no kidney replacement therapy code within three months of the UKRR start date.

### Discrepancies between primary care, secondary care, and UKRR data

We found that many people were identified as recipients of kidney replacement therapy (particularly dialysis) in primary and secondary care data only, but only some were potentially accounted for by the known reasons that people receiving kidney replacement therapy are not included in UKRR cohorts. Firstly, the prevalent UKRR cohorts did not include people who stopped kidney replacement therapy by the prevalent date (eg, because of recovery or conservative management); these patients would be included in primary and secondary care cohorts. Secondly, the UKRR database operates on an annual basis and people could be present in one year and not included in a later year (eg, patients who received a kidney transplant who might have moved away temporarily and were cared for only by their primary care doctor on their return). Thirdly, border effects might exist in that only data for English renal centres were submitted to OpenSAFELY, so people residing in England but treated at a Welsh or Scottish renal centre would not be in the UKRR cohort but could be in primary or secondary care cohorts if they were registered with an OpenSAFELY-TPP practice. From UKRR data, we estimate that, in total, these groups would account for <1600 people in the study cohort.

Nationally, more people are registered at English general practices than are in the population,^18^ so over-registration (eg, of people who have moved abroad) might contribute to the differences seen. The UKRR definition of incidence of kidney replacement therapy excludes those who recover kidney function before 90 days of dialysis treatment, and those who die after starting acute dialysis. A previous UKRR analysis estimated that 20% of people who ever start dialysis are still alive but no longer receiving kidney replacement therapy at 90 days.^19^ Patients recovering within 90 days who are still alive will continue to be considered part of the kidney replacement therapy population in primary and secondary care, although they would not be identified as an incident patient in the UKRR study population. Also, acute dialysis can be delivered by intensive care staff without involvement of renal care. We believe that acute dialysis is the main reason for the discrepancy between UKRR and secondary care data. The 90 day requirement might also contribute to the discrepancy in start dates, because if a patient receives acute dialysis and continues beyond 90 days to require chronic dialysis, their start date in UKRR data is considered to be when they started acute dialysis. Start dates might not be determined the same way in primary care data.

We found no primary or secondary care codes whose exclusion would substantially improve the positive predictive value without a decrease in sensitivity. Some codes, along with critical care data, however, could be used to flag the patient record for further investigation. In particular, the acquired arteriovenous fistula code indicates dialysis preparation but not actual dialysis; the transplant nephrectomy code was less common among people also in the UKRR cohorts and could be a miscoding of nephrectomy. Among transplant codes, live donor renal transplant and donor renal transplantation might have been entered for the donor rather than the recipient. The proportion of secondary care inpatient episodes featuring some critical care was higher in people not in the UKRR cohort for the codes for dialysis not elsewhere classified and haemodialysis not elsewhere classified, possibly indicating acute dialysis. In the primary care data, restriction based on estimated glomerular filtration rate measurements can reduce the number of people incorrectly identified as receiving chronic dialysis but with limited use for transplant or overall kidney replacement therapy.

### Strengths and weaknesses of this study

Our study linked UKRR data with primary and secondary care data at a population level, inclusive of all ages, by using the secure OpenSAFELY platform. This approach allowed validation of primary and secondary care coding in both directions, rather than being limited to assessment of sensitivity through linkage of the UKRR cohort only. The UKRR has been established for more than 25 years, providing in-depth data with complete UK coverage for all adults and children receiving chronic kidney replacement therapy, making it a unique resource for kidney medicine and facilitating analysis that is not possible in other clinical areas. Data undergo extensive validation and cleaning, and thus UKRR data can be considered a gold standard for defining incident and prevalent chronic kidney replacement therapy cohorts.

A key limitation of our study is that the analysis was restricted to people in the OpenSAFELY-TPP database. For the whole patient population, these data have been shown to be reasonably representative of the English population,^17^ but we found differences compared with the UKRR prevalent cohort. This finding is in part because of the disparities in the cohorts identified by the three data sources (as we set out to describe) but also might be because London has a high prevalence of kidney replacement therapy^20^ but is under-represented in the OpenSAFELY-TPP database.

Previous work by Iwagami et al^21^ compared estimated population prevalence in the Clinical Practice Research Datalink with published UKRR data from 2014, and found a similar estimated prevalence of kidney replacement therapy in the two sources. Considering the low prevalence of kidney replacement therapy in the population (0.05%^22^), discrepancies in the UKRR and primary care kidney replacement therapy cohorts in our study would not have corresponded to notable changes in prevalence. Iwagami et al reported a lower prevalence of haemodialysis in the Clinical Practice Research Datalink compared with UKRR, whereas we found larger numbers of patients receiving dialysis in the primary care data. Our study looked at haemodialysis and peritoneal dialysis together and included more dialysis codes. Also, coding practices and accuracy might have changed since 2014, although no other studies exist to confirm this. Our results are in contrast with findings from international systematic reviews^23 24^ on the accuracy of coding of chronic kidney disease in electronic health records, where studies generally had poor sensitivity but good specificity and reasonable positive predictive values. This difference suggests that the wider body of work on the validity of using administrative data for chronic kidney disease^23–27^ is not applicable, and supports the need for further studies looking specifically at kidney replacement therapy.

This linkage was done to understand how previous work on covid-19 based on only primary and secondary care data to identify people receiving kidney replacement therapy might have been affected by misclassification. In this study, we restricted the primary and secondary care definitions to the presence of one of a list of codes indicating kidney replacement therapy in a patient's history, rather than using combinations or exclusions of codes, or requiring codes to be present multiple times to indicate chronic dialysis. This method reflected the approach taken in previous studies of patients receiving kidney replacement therapy in OpenSAFELY, itself based on previous work in other sources of primary care data, but the positive predictive value might be increased while maintaining sensitivity if more complex definitions were applied.

Based on our findings, in general, analyses that do not use UKRR data cannot reliably distinguish between people who have had acute dialysis from those who remain on chronic kidney replacement therapy. More than a third of people starting dialysis are given an acute code in UKRR data, and nearly a quarter of these will still be receiving kidney replacement therapy on day 90 and thus considered to be on chronic dialysis.^19^ Depending on the question, the distinction between acute and chronic dialysis is perhaps not important, especially in terms of identifying risk factors for poor outcomes related to covid-19 disease. For chronic kidney replacement therapy, particularly if correct start dates are needed, registry data are required. For researchers interested in whether people have ever required any form of kidney replacement therapy (eg, as a baseline risk factor for other outcomes), then a dataset based on primary and secondary care data only could be considered sufficient. We found that most people incorrectly identified as prevalent recipients of kidney replacement therapy in primary care data had reduced kidney function based on their latest estimated glomerular filtration rate. For previous studies of populations receiving kidney replacement therapy based on OpenSAFELY, some misclassification across stages of chronic kidney disease could have occurred, but if anything, the broader definition would likely have led to attenuated findings.

### Policy implications and interpretation

Primary and secondary care electronic health records were used during the covid-19 pandemic to identify clinically vulnerable people and communicate shielding advice. Accurate and prompt coding of people with immunosuppression and other high risk conditions is needed to ensure these patients are adequately protected in future pandemics. Some patients who were eligible for interventions, such as vaccination or antiviral treatment, may not have been identified in a timely manner by primary and secondary care codes, as demonstrated by the analysis of kidney replacement therapy start dates. Communication with patients and care providers may therefore have been suboptimal. Accurate and prompt coding of kidney replacement therapy is needed to ensure that clinically vulnerable groups are adequately protected in future pandemics.

Evaluation of short term outcomes of covid-19 disease is perhaps less relevant in children because of the comparably lower risk of adverse outcomes, but these findings suggest linkage of UKRR data is necessary to monitor vaccination trends and long term outcomes after infection in this cohort.^28^ Children living with kidney disease have a substantial disease burden of treatment throughout their lives, with reduced life years compared with their peers,^29^ and identifying this cohort and monitoring their care is therefore imperative. Poor coding in primary and secondary care data is concerning. We saw variation in the accuracy of coding across age ranges, as well as by ethnic group and index of multiple deprivation, limiting the ability to provide an equitable health service across the population. Coding is often carried out by inexperienced staff, but inaccuracies can have substantial implications for local resources.^30 31^

Outside of the context of covid-19, obtaining linked data can be challenging with additional resource and governance requirements. Our analyses can help in clarifying whether routine primary or secondary care electronic health records for a particular project would suffice, thus saving resources if UKRR data are not required. When only routine secondary and primary care data are used, as is typical in pharmaco-epidemiology studies, we showed that linkage to a kidney registry is required to accurately identify starting dates for those who require long term dialysis or kidney transplantation. On the other hand, our work showed the extent of acute kidney care that is performed (and not reported in registries of chronic kidney failure), which is particularly relevant for settings where financing of kidney services is driven only by chronic need. More generally, this study highlighted the value of linking registry data to routine electronic health records with implications beyond kidney medicine, because it adds to a growing body of work demonstrating similar benefits in a range of clinical areas, such as cardiovascular events,^5 6 32^ cancer,^33^ and diabetes.^34^

### Conclusions

Linkage with UKRR kidney replacement therapy data facilitated more accurate identification of incident and prevalent cohorts receiving kidney replacement therapy than was achieved with only electronic health records. Codes used in primary and secondary care data only missed a small proportion of prevalent patients receiving kidney replacement therapy. Codes also identified many patients not receiving chronic kidney replacement therapy in UKRR data, particularly dialysis codes. This study also showed that new patients starting dialysis for the first time are not identified promptly by primary care codes leading to a delay in receiving timely interventions for patients with immunosuppression. Poor coding also has implications for any patient care, including resource planning, that relies on accurate recording of kidney replacement therapy in primary and secondary care data.

10.1136/bmjmed-2023-000807.supp2 Supplementary data

## Data Availability

No data are available. Detailed pseudonymised patient data are potentially re-identifiable and therefore not shared. Access to the underlying identifiable and potentially re-identifiable pseudonymised electronic health record data is tightly governed by various legislative and regulatory frameworks, and restricted by best practice. The data in OpenSAFELY are drawn from general practice data across England where TPP is the data processor. TPP developers initiate an automated process to create pseudonymised records in the core OpenSAFELY database, which are copies of key structured data tables in the identifiable records. These are linked onto key external data resources that have also been pseudonymised through SHA-512 one way hashing of NHS numbers with a shared salt. Bennett Institute for Applied Data Science developers and principal investigators holding contracts with NHS England have access to the OpenSAFELY pseudonymised data tables as needed to develop the OpenSAFELY tools. These tools in turn enable researchers with OpenSAFELY data access agreements to write and execute code for data management and data analysis without direct access to the underlying raw pseudonymised patient data, and to review the outputs of this code. All code for the full data management pipeline—from raw data to completed results for this analysis—and for the OpenSAFELY platform as a whole is available for review at github.com/OpenSAFELY. The UK Renal Registry (UKRR) data are collected to enable the UK Kidney Association to perform its audit function, but some data are also available for research and local audit. Staff based in NHS or academic institutions can apply for access for the purposes of conducting audit or research which improves the care and outcomes of patients with kidney disease. Details on how to access UKRR data are available at https://ukkidney.org/audit-research/how-access-data/ukrr-data.
