# Supplementary material for: Identification of patients undergoing chronic kidney replacement therapy in primary and secondary care data: validation study based on OpenSAFELY and UK Renal Registry
Source: BMJ Med. 2024 Apr 18;3(1):e000807. doi: 10.1136/bmjmed-2023-000807 (PMC11029353; doi:10.1136/bmjmed-2023-000807)

Supplementary information

S1 Codelists

S1A Primary care codelists
Existing codelists published on opencodelists.org
KRT https://www.opencodelists.org/codelist/opensafely/renal-replacement-therapy/2020-04-14/
Dialysis
https://www.opencodelists.org/codelist/opensafely/dialysis/3ce108ac/
Transplant
https://www.opencodelists.org/codelist/opensafely/kidney-transplant/2020-07-15/
CKD
https://www.opencodelists.org/codelist/primis-covid19-vacc-uptake/ckd15/v.1.5.3/
https://www.opencodelists.org/codelist/opensafely/chronic-kidney-disease-snomed/2020-04-14/

Santhakumaran S, *et al. BMJMED* 2024; 3:e000807. doi: 10.1136/bmjmed-2023-000807

Table S1B - Secondary care codelists

Modality	Coding system	Code	Description
Dialysis	ICD-10	T824	Mechanical complication of vascular dialysis catheter
		Y602	Unintentional cut/puncture/perforation or haemorrhage during surgical and medical care - during dialysis or other perfusion
		Y612	Foreign object accidentally left in body during medical care - during kidney dialysis or other perfusion
		Y622	Failure of sterile precautions during surgical or medical care- during kidney dialysis or other perfusion
		Y841	Other medical procedures as the cause of abnormal reactions of the patient, or of later complication, without mention of misadventure at the time of the procedure - kidney dialysis
		Z49	Care involving dialysis
		Z49.31	Encounter for adequacy testing for haemodialysis
		Z49.32	Encounter for adequacy testing for peritoneal dialysis
		Z491	Extracorporeal dialysis
		Z492	Other dialysis
		Z493	Encounter for adequacy testing for dialysis
		Z992	Dependence on renal dialysis
Dialysis	OPCS-4	X40	Dialysis NEC
		X401	Renal dialysis
		X402	Peritoneal dialysis NEC
		X403	Haemodialysis NEC
		X405	Automated peritoneal dialysis
		X406	Continuous ambulatory peritoneal dialysis
		X408	Other specified compensation for renal failure
		X409	Unspecified compensation for renal failure
		X41	Placement of ambulatory apparatus for compensation for renal failure
		X411	Insertion of ambulatory peritoneal dialysis catheter

Modality	Coding system	Code	Description
		X418	Other specified
		X419	Unspecified
		X42	Placement of other apparatus for compensation for renal failure
		X421	Insertion of temporary peritoneal dialysis catheter
		X428	Other specified
		X429	Unspecified
Transplant	ICD-10	Z940	Kidney transplant status
Transplant	OPCS-4	M01	Transplantation of kidney
		M012	Allotransplantation of kidney from live donor
		M013	Allotransplantation of kidney from cadaver NEC
		M014	Allotransplantation of kidney from cadaver heart beating
		M015	Allotransplantation of kidney from cadaver heart non-beating
		M018	Other specified transplantation of kidney
		M019	Unspecified transplantation of kidney
		M084	Exploration of transplanted kidney
		M17	Interventions associated with transplantation of kidney
		M174	Post-transplantation of kidney examination - recipient
		M178	Other specified interventions associated with transplantation of kidney
		M179	Unspecified interventions associated with transplantation of kidney
Additional KRT*	ICD-10	T861	Kidney transplant failure and rejection
Additional KRT*	OPCS-4	M023	Bilateral nephrectomy
		M026	Excision of rejected transplanted kidney
		M027	Excision of transplanted kidney NEC

Santhakumaran S, *et al. BMJMED* 2024; 3:e000807. doi: 10.1136/bmjmed-2023-000807

Modality	Coding system	Code	Description
		X412	Removal of ambulatory peritoneal dialysis catheter

* Secondary care KRT ICD-10 and OPCS-4 codelists include the codes from the corresponding dialysis and transplant codelists, plus the additional codes listed here

Santhakumaran S, *et al. BMJMED* 2024; 3:e000807. doi: 10.1136/bmjmed-2023-000807

Table S2 – Comparative UKRR data for the whole of England

	Receiving KRT at start of 2020	Receiving dialysis at start of 2020	Receiving transplant at start of 2020	Receiving KRT during 2020
	N (%)	N (%)	N (%)	N (%)
Total	57,545	25,157	32,388	6,283
Age				
18-39	7,458 (13)	2,045 (8)	5,413 (17)	692 (11)
40-49	8,265 (14)	2,393 (10)	5,872 (18)	669 (11)
50-59	13,597 (24)	4,600 (18)	8,997 (28)	1,255 (20)
60-69	13,062 (23)	5,566 (22)	7,496 (23)	1,448 (23)
70-79	10,531 (18)	6,419 (26)	4,112 (13)	1,538 (24)
80+	4,632 (8)	4,134 (16)	498 (2)	681 (11)
Median (IQR)	60 (49,71)	66 (55,77)	56 (45,65)	64 (52,74)
Sex				
Male	22,268 (39)	9,581 (38)	12,687 (39)	2,217 (35)
Female	35,277 (61)	15,576 (62)	19,701 (61)	4,066 (65)
Ethnicity				
Asian	8,235 (14)	3,763 (15)	4,472 (14)	860 (14)
Black	5,056 (9)	2,936 (12)	2,120 (7)	493 (8)
Mixed	824 (1)	393 (2)	431 (1)	85 (1)
Other	1,075 (2)	506 (2)	569 (2)	120 (2)
White	41,389 (72)	16,873 (67)	24,516 (76)	4,219 (67)
Missing	966(2)	68 86 (3)	280(1)	506 (8)
IMD quintile				
1 - most deprived	13,685 (24)	7,065 (28)	6,620 (20)	1,562 (25)
2	12,956 (23)	6,002 (24)	6,954 (21)	1,419 (23)
3	11,447 (20)	4,872 (19)	6,575 (20)	1,227 (20)
4	10,240 (18)	3,963 (16)	6,277 (19)	1,125 (18)
5 - least deprived	9,153 (16)	3,233 (13)	5,920 (18)	945 (15)
Missing	64(0)	22(0)	42(0)	5 (0)
Region				

East of England	4,653 (8)	2,139 (9)	2,514 (8)	577 (9)
London	14,818 (26)	6,557 (26)	8,261 (26)	1,490 (24)
Midlands	11,053 (19)	5,392 (21)	5,661 (17)	1,307 (21)
North East	2,996 (5)	1,238 (5)	1,758 (5)	323 (5)
North West	6,474 (11)	2,632 (10)	3,842 (12)	661 (11)
South East	6,914 (12)	2,674 (11)	4,240 (13)	805 (13)
South West	4,861 (8)	2,058 (8)	2,803 (9)	514 (8)
Yorkshire & Humber	5,776 (10)	2,467 (10)	3,309 (10)	606 (10)

Santhakumaran S, *et al. BMJMED* 2024; 3:e000807. doi: 10.1136/bmjmed-2023-000807

BMJ Publishing Group Limited (BMJ) disclaims all liability and responsibility arising from any reliance placed on this supplemental material which has been supplied by the author(s)

Table S3 - Characteristics of the study population by data source and modality. All n rounded to the nearest 5. For primary and secondary care total KRT may exceed the sum of dialysis and transplant as some KRT codes do not indicate modality.

	KRT recipient at start of 2020			Dialysis recipient at start of 2020			Transplant recipient at start of 2020			Starting KRT during 2020		
	UKRR	PC	SC	UKRR	PC	SC	UKRR	PC	SC	UKRR	PC	SC
	N (%)	N (%)	N (%)	N (%)	N (%)	N (%)	N (%)	N (%)	N (%)	N (%)	N (%)	N (%)
Total	22,815	26,750	32,465	9,780	13,985	16,780	13,035	12,565	14,605	2,475	2,880	8,730
Age												
18-39	2,975 (13)	3,655 (14)	4,040 (12)	790 (8)	1,465 (10)	1,345 (8)	2,185 (17)	2,155 (17)	2,455 (17)	250 (10)	290 (10)	990 (11)
40-49	3,135 (14)	3,645 (14)	4,125 (13)	865 (9)	1,390 (10)	1,420 (8)	2,270 (17)	2,220 (18)	2,530 (17)	270 (11)	315 (11)	1,095 (13)
50-59	5,335 (23)	6,005 (22)	6,855 (21)	1,695 (17)	2,530 (18)	2,630 (16)	3,640 (28)	3,425 (27)	3,935 (27)	525 (21)	550 (19)	1,835 (21)
60-69	5,185 (23)	5,895 (22)	7,370 (23)	2,190 (22)	3,005 (21)	3,750 (22)	2,995 (23)	2,845 (23)	3,385 (23)	570 (23)	595 (21)	2,075 (24)
70-79	4,255 (19)	5,020 (19)	6,835 (21)	2,535 (26)	3,335 (24)	4,715 (28)	1,720 (13)	1,655 (13)	2,000 (14)	620 (25)	695 (24)	2,015 (23)
80+	1,925 (8)	2,535 (9)	3,235 (10)	1,705 (17)	2,270 (16)	2,920 (17)	220 (2)	265 (2)	300 (2)	230 (9)	435 (15)	715 (8)
Median (IQR), years	59 (49,71)	60 (48,71)	61 (49,72)	67 (55,77)	65 (53,76)	68 (55,77)	55 (45,65)	55 (45,65)	56 (45,65)	63 (52,73)	65 (52,75)	62 (50,72)
Sex												
Female	8,675 (38)	10,730 (40)	12,830 (40)	3,615 (37)	5,640 (40)	6,365 (38)	5,060 (39)	5,005 (40)	6,035 (41)	835 (34)	1,060 (37)	3,165 (36)
Male	14,140 (62)	16,020 (60)	19,635 (60)	6,165 (63)	8,345 (60)	10,415 (62)	7,975 (61)	7,560 (60)	8,570 (59)	1,640 (66)	1,820 (63)	5,565 (64)
Ethnicity												
White	18,205 (80)	21,745 (81)	26,755 (82)	7,515 (77)	11,360 (81)	13,990 (83)	10,690 (82)	10,220 (81)	11,950 (82)	1,970 (80)	2,410 (84)	7,330 (84)
Mixed	270 (1)	310 (1)	365 (1)	145 (1)	170 (1)	180 (1)	125 (1)	140 (1)	160 (1)	30 (1)	25 (1)	105 (1)
South Asian	2,865 (13)	2,995 (11)	3,520 (11)	1,355 (14)	1,500 (11)	1,670 (10)	1,510 (12)	1,475 (12)	1,700 (12)	315 (13)	280 (10)	795 (9)
Black	1,075 (5)	1,125 (4)	1,280 (4)	610 (6)	665 (5)	705 (4)	465 (4)	450 (4)	510 (3)	110 (4)	110 (4)	310 (4)
Other	375 (2)	410 (2)	450 (1)	145 (1)	185 (1)	185 (1)	230 (2)	225 (2)	245 (2)	30 (1)	35 (1)	115 (1)
Missing	25 (0)	160 (1)	85 (0)	15 (0)	105 (1)	50 (0))	10 (0)	55 (0)	35 (0)	≤7 (0)	10 (0)	75 (1)
IMD quintile												
1 - most deprived	5,495 (24)	5,995 (22)	7,445 (23)	2,710 (28)	3,490 (25)	4,235 (25)	2,560 (20)	2,465 (20)	2,955 (20)	595 (24)	690 (24)	2,045 (23)
2	4,880 (21)	5,600 (21)	6,845 (21)	2,195 (22)	2,985 (21)	3,615 (22)	2,680 (21)	2,580 (21)	2,990 (20)	550 (22)	625 (22)	1,865 (21)
3	4,710 (20)	5,530 (21)	6,800 (21)	1,955 (20)	2,825 (20)	3,475 (21)	2,750 (21)	2,665 (21)	3,110 (21)	530 (21)	600 (21)	1,860 (21)
4	4,070 (18)	4,965 (19)	5,910 (18)	1,520 (16)	2,455 (18)	2,870 (17)	2,550 (20)	2,470 (20)	2,860 (20)	425 (17)	490 (17)	1,570 (18)
5 - least deprived	3,530 (15)	4,245 (16)	4,960 (15)	1,250 (13)	2,005 (14)	2,325 (14)	2,280 (17)	2,195 (17)	2,460 (17)	325 (13)	410 (14)	1,250 (14)
Missing	360 (2)	420 (2)	510 (2)	150 (2)	230 (2)	260 (2)	210 (2)	190 (2)	235 (2)	45 (2)	60 (2)	145 (2)

Diabetes	8,770 (38)	9,510 (36)	12,285 (38)	4,410 (45)	5,410 (39)	7,480 (45)	4,360 (33)	4,045 (32)	4,450 (30)	1,195 (48)	1,310 (45)	3,165 (36)
Hypertension	19,610 (86)	21,475 (80)	24,810 (76)	8,530 (87)	11,020 (79)	12,710 (76)	11,080 (85)	10,285 (82)	11,195 (77)	2,015 (81)	2,235 (77)	5,370 (61)
Region												
North East	1,130 (5)	1,270 (5)	1,750 (5)	525 (5)	665 (5)	910 (5)	605 (5)	605 (5)	830 (6)	120 (5)	130 (5)	440 (5)
North West	1,925 (8)	2,315 (9)	2,760 (9)	745 (8)	1,200 (9)	1,580 (9)	1,180 (9)	1,095 (9)	1,040 (7)	200 (8)	245 (9)	680 (8)
Yorkshire & Humber	3,375 (15)	4,140 (15)	4,695 (14)	1,390 (14)	2,355 (17)	2,075 (12)	1,985 (15)	1,745 (14)	2,460 (17)	325 (13)	450 (16)	1,300 (15)
East Midlands	3,970 (17)	4,705 (18)	5,660 (17)	1,690 (17)	2,355 (17)	2,850 (17)	2,280 (18)	2,315 (18)	2,635 (18)	445 (18)	495 (17)	1,595 (18)
West Midlands	1,150 (5)	1,255 (5)	1,675 (5)	675 (7)	795 (6)	1,085 (6)	475 (4)	460 (4)	540 (4)	110 (4)	145 (5)	470 (5)
East of England	5,105 (22)	5,900 (22)	7,145 (22)	2,145 (22)	2,925 (21)	3,870 (23)	2,960 (23)	2,910 (23)	3,000 (21)	575 (23)	640 (22)	2,015 (23)
London	1,770 (8)	1,925 (7)	2,270 (7)	840 (9)	940 (7)	1,215 (7)	930 (7)	975 (8)	905 (6)	190 (8)	170 (6)	565 (6)
South East	1,325 (6)	1,620 (6)	2,100 (6)	545 (6)	890 (6)	965 (6)	780 (6)	715 (6)	1,095 (8)	170 (7)	175 (6)	590 (7)
South West	3,060 (13)	3,605 (13)	4,385 (14)	1,230 (13)	1,850 (13)	2,215 (13)	1,830 (14)	1,740 (14)	2,085 (14)	320 (13)	420 (15)	1,050 (12)
Missing	15 (0)	≤7 (0)	20 (0)	≤7 (0)	≤7 (0)	10 (0)	≤7 (0)	≤7 (0)	10 (0)	0 (0)	0 (0)	0 (0)

Santhakumaran S, *et al. BMJMED* 2024; 3:e000807. doi: 10.1136/bmjmed-2023-000807

Table S4 Agreement by demographic characteristics for prevalent patients receiving KRT at the start of 2020

Category	UKRR+PC+SC	UKRR+PC	UKRR-SC	PC+SC	UKRR only	PC only	SC only
	N (%)	N (%)	N (%)	N (%)	N (%)	N (%)	N (%)
Age							
18-39	2,555 (51)	265 (5)	145 (3)	145 (3)	0 (0)	685 (14)	1,195 (24)
40-49	2,640 (53)	320 (6)	160 (3)	170 (3)	15 (0)	510 (10)	1,155 (23)
50-59	4,515 (56)	475 (6)	325 (4)	250 (3)	25 (0)	765 (9)	1,765 (22)
60-69	4,360 (51)	360 (4)	430 (5)	345 (4)	35 (0)	830 (10)	2,235 (26)
70-79	3,550 (45)	175 (2)	505 (6)	385 (5)	30 (0)	915 (11)	2,400 (30)
80+	1,495 (36)	90 (2)	315 (8)	200 (5)	25 (1)	750 (18)	1,225 (30)
Sex							
Female	7,405 (48)	560 (4)	665 (4)	630 (4)	40 (0)	2,140 (14)	4,125 (27)
Male	11,710 (51)	1,125 (5)	1,205 (5)	865 (4)	95 (0)	2,320 (10)	5,850 (25)
Ethnicity							
White	15,235 (47)	1,420 (4)	1,435 (4)	1,280 (4)	115 (0)	3,645 (11)	8,740 (28)
Mixed	240 (56)	10 (2)	15 (4)	20 (5)	0 (0)	50 (11)	100 (21)
South Asian	2,435 (61)	135 (3)	280 (7)	125 (3)	15 (0)	280 (7)	680 (17)
Black	885 (60)	80 (5)	105 (7)	50 (3)	≤7 (0)	95 (7)	230 (16)
Other	310 (58)	35 (7)	25 (5)	20 (4)	≤7 (0)	60 (10)	115 (19)
IMD quintile							
1 - most deprived	4,385 (50)	305 (3)	550 (6)	345 (4)	35 (0)	970 (11)	2,165 (25)
2	4,090 (51)	320 (4)	435 (5)	315 (4)	30 (0)	875 (11)	2,005 (25)
3	3,970 (49)	360 (4)	340 (4)	345 (4)	35 (0)	860 (11)	2,140 (27)
4	3,440 (48)	345 (5)	270 (4)	265 (4)	20 (0)	915 (13)	1,940 (27)
5 - least deprived	2,945 (49)	335 (6)	235 (4)	215 (4)	20 (0)	750 (12)	1,565 (26)
Diabetes	7,305 (51)	490 (3)	920 (6)	570 (4)	55 (0)	1,145 (8)	3,845 (27)
Hypertension	16,530 (57)	1,435 (5)	1,535 (5)	1070 (4)	115 (0)	2,440 (8)	5,675 (20)
Region							
North East	985 (50)	30 (2)	110 (6)	90 (5)	≤7 (0)	175 (9)	570 (29)
North West	1,545 (45)	220 (6)	145 (4)	125 (4)	10 (0)	425 (12)	940 (28)
Yorkshire & Humber	2,955 (52)	200 (4)	200 (4)	205 (4)	15 (0)	780 (14)	1,330 (23)
East Midlands	3,340 (49)	320 (5)	285 (4)	270 (4)	25 (0)	775 (11)	1,765 (26)
West Midlands	935 (49)	70 (4)	135 (7)	80 (4)	≤7 (0)	170 (9)	520 (27)
East of England	4,090 (47)	480 (6)	490 (6)	360 (4)	40 (0)	965 (11)	2,195 (25)

London	1,435 (53)	135 (5)	185 (7)	70 (3)	10 (0)	280 (10)	580 (22)
South East	1,160 (47)	60 (2)	95 (4)	110 (4)	10 (0)	285 (12)	735 (30)
South West	2,660 (52)	170 (3)	220 (4)	180 (3)	10 (0)	590 (11)	1,325 (26)

Santhakumaran S, *et al. BMJMED* 2024; 3:e000807. doi: 10.1136/bmjmed-2023-000807

Table S5 Sensitivity and positive predictive value of primary and secondary care definitions (taking UKRR as the gold standard) by demographic characteristics, for prevalent dialysis and transplant cohorts at the start of 2020

	Dialysis recipient at the start of 2020				Transplant recipient at the start of 2020			
Category	Primary care		Secondary care		Primary care		Secondary care	
	Sensitivity (95% CI)	PPV (95% CI)	Sensitivity (95% CI)	PPV (95% CI)	Sensitivity (95% CI)	PPV (95% CI)	Sensitivity (95% CI)	PPV (95% CI)
All patients	77 (76,78)	54 (53,55)	84 (83,85)	49 (48,50)	84 (83,85)	87 (86,88)	84 (83,85)	75 (74,76)
Age								
18-39	75 (72,78)	40 (37,43)	71 (68,74)	41 (37,45)	86 (85,87)	87 (86,88)	81 (79,83)	73 (71,75)
40-49	74 (71,77)	46 (43,49)	73 (70,76)	45 (41,49)	85 (84,86)	87 (86,88)	83 (81,85)	74 (72,76)
50-59	76 (74,78)	51 (49,53)	78 (76,80)	50 (47,53)	84 (83,85)	89 (88,90)	83 (82,84)	77 (76,78)
60-69	76 (74,78)	56 (54,58)	83 (81,85)	49 (47,51)	84 (83,85)	88 (87,89)	85 (84,86)	76 (74,78)
70-79	78 (76,80)	59 (57,61)	91 (90,92)	49 (47,51)	83 (81,85)	86 (84,88)	90 (89,91)	77 (75,79)
80+	79 (77,81)	60 (58,62)	94 (93,95)	55 (53,57)	84 (79,89)	70 (64,76)	86 (81,91)	63 (56,70)
Sex								
Female	78 (77,79)	50 (49,51)	84 (83,85)	48 (46,50)	85 (84,86)	86 (85,87)	87 (86,88)	73 (72,74)
Male	76 (75,77)	56 (55,57)	84 (83,85)	50 (49,51)	84 (83,85)	88 (87,89)	83 (82,84)	77 (76,78)
Ethnicity								
White	77 (76,78)	51 (50,52)	85 (84,86)	46 (45,47)	84 (83,85)	88 (87,89)	84 (83,85)	75 (74,76)
Mixed	79 (72,86)	68 (61,75)	76 (69,83)	61 (52,70)	92 (87,97)	79 (72,86)	88 (82,94)	66 (57,75)
South Asian	74 (72,76)	66 (64,68)	83 (81,85)	67 (64,70)	83 (81,85)	85 (83,87)	87 (85,89)	77 (75,79)
Black	77 (74,80)	71 (68,74)	81 (78,84)	70 (66,74)	81 (77,85)	84 (81,87)	82 (79,85)	75 (71,79)
Other	79 (72,86)	61 (54,68)	83 (77,89)	63 (54,72)	83 (78,88)	86 (81,91)	83 (78,88)	78 (72,84)
IMD quintile								
1 - most deprived	75 (73,77)	58 (56,60)	84 (83,85)	54 (52,56)	83 (82,84)	86 (85,87)	86 (85,87)	74 (72,76)
2	77 (75,79)	56 (54,58)	85 (84,86)	51 (49,53)	84 (83,85)	87 (86,88)	85 (84,86)	76 (74,78)
3	77 (75,79)	53 (51,55)	83 (81,85)	47 (45,49)	84 (83,85)	87 (86,88)	85 (84,86)	75 (73,77)
4	79 (77,81)	49 (47,51)	85 (83,87)	45 (42,48)	85 (84,86)	88 (87,89)	84 (83,85)	74 (72,76)
5 - least deprived	78 (76,80)	48 (46,50)	84 (82,86)	45 (42,48)	86 (85,87)	89 (88,90)	82 (80,84)	76 (74,78)
Diabetes	76 (75,77)	62 (61,63)	88 (87,89)	52 (50,54)	85 (84,86)	91 (90,92)	86 (85,87)	84 (83,85)
Hypertension	77 (76,78)	60 (59,61)	84 (83,85)	56 (55,57)	84 (83,85)	91 (90,92)	85 (84,86)	84 (83,85)
Region								
North East	73 (69,77)	58 (54,62)	88 (85,91)	51 (46,56)	91 (89,93)	91 (89,93)	96 (94,98)	70 (66,74)
North West	77 (74,80)	48 (45,51)	88 (86,90)	42 (38,46)	83 (81,85)	89 (87,91)	74 (72,76)	83 (81,85)
Yorkshire & Humber	81 (79,83)	48 (46,50)	77 (75,79)	51 (48,54)	77 (75,79)	88 (86,90)	89 (88,90)	72 (70,74)
East Midlands	79 (77,81)	57 (55,59)	82 (80,84)	49 (46,52)	90 (89,91)	89 (88,90)	84 (82,86)	73 (71,75)
West Midlands	75 (72,78)	63 (60,66)	90 (88,92)	56 (52,60)	78 (74,82)	82 (78,86)	83 (80,86)	73 (69,77)
East of England	73 (71,75)	54 (52,56)	85 (83,87)	47 (45,49)	85 (84,86)	86 (85,87)	80 (79,81)	79 (77,81)
London	74 (71,77)	65 (62,68)	85 (83,87)	58 (54,62)	84 (82,86)	80 (77,83)	80 (77,83)	81 (78,84)
South East	79 (76,82)	49 (46,52)	75 (71,79)	42 (37,47)	81 (78,84)	88 (86,90)	93 (91,95)	66 (63,69)
South West	79 (77,81)	53 (51,55)	91 (89,93))	50 (47,53)	85 (83,87)	90 (89,91)	88 (87,89)	78 (76,80)

Santhakumaran S, *et al. BMJMED* 2024; 3:e000807. doi: 10.1136/bmjmed-2023-000807

Table S6 Sensitivity and positive predictive value for 2020 incident cohorts

	Starting KRT during 2020		
	Any KRT	Dialysis	Transplant
Primary care			
Sensitivity (95% CI)	52 (50,54)	49 (47,51)	79 (73,85)
PPV (95% CI)	45 (43,47)	44 (42,46)	38 (33,43)
Secondary care			
Sensitivity (95% CI)	68 (66,70)	65 (63,67)	42 (34,50)
PPV (95% CI)	19 (17,21)	22 (20,24)	3 (0,7)

Santhakumaran S, *et al. BMJMED* 2024; 3:e000807. doi: 10.1136/bmjmed-2023-000807

Figure S1 Most common primary/secondary care codes for people incident/prevalent to KRT in primary/secondary care data, but who were not in the UKRR, and the frequency amongst people who are also in the UKRR for comparison. All n rounded to the nearest 5 and n ≤ 7 not shown.

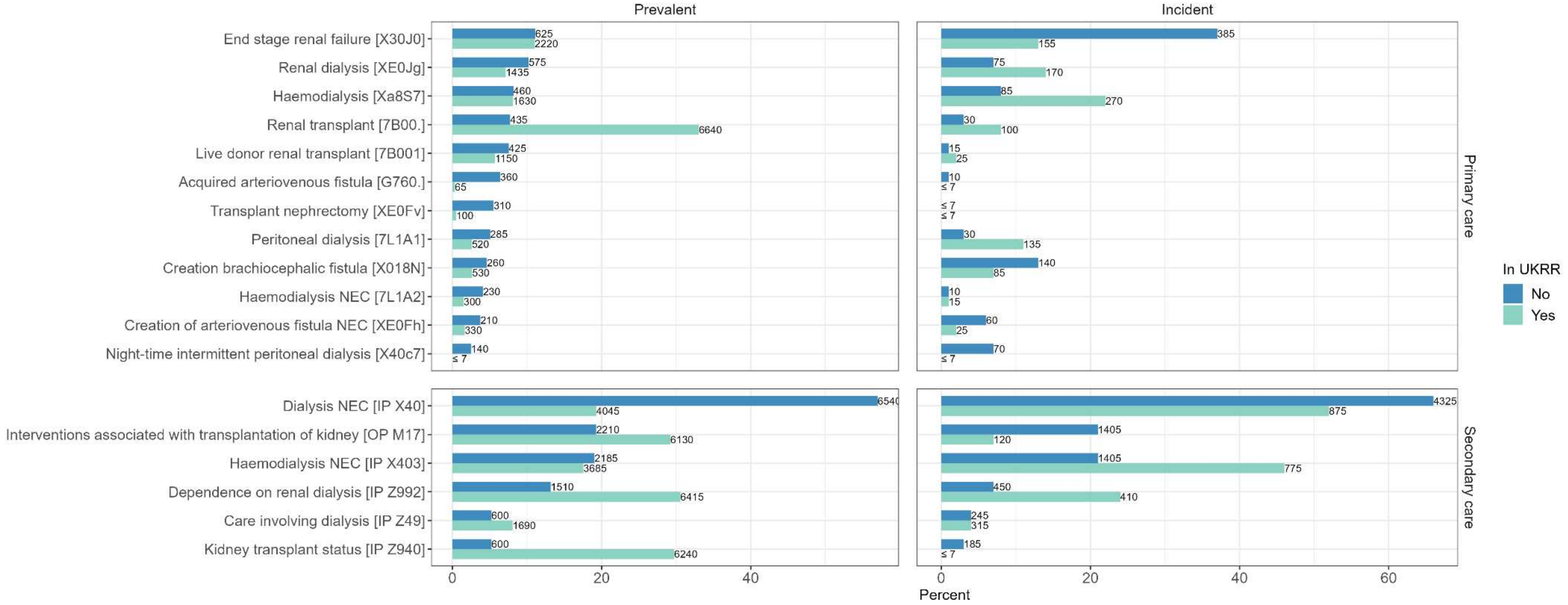

Figure S2 Euler diagrams showing agreement for under 18 prevalent cohorts on 1st January 2020 for a) all KRT, b) dialysis and c) transplant. All n rounded to the nearest 5. Values ≤ 7 are shown as 7.

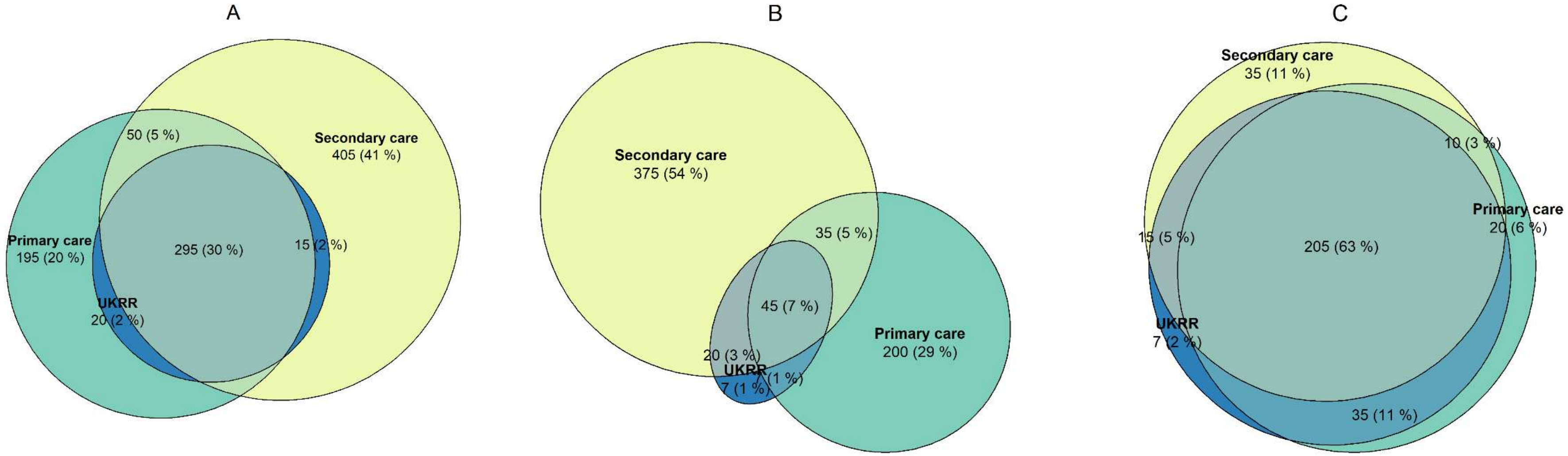

Figure S3 Euler diagrams showing agreement for adult prevalent cohorts on a) 1st January 2021 and b) 1st January 2022. All n rounded to the nearest 5.

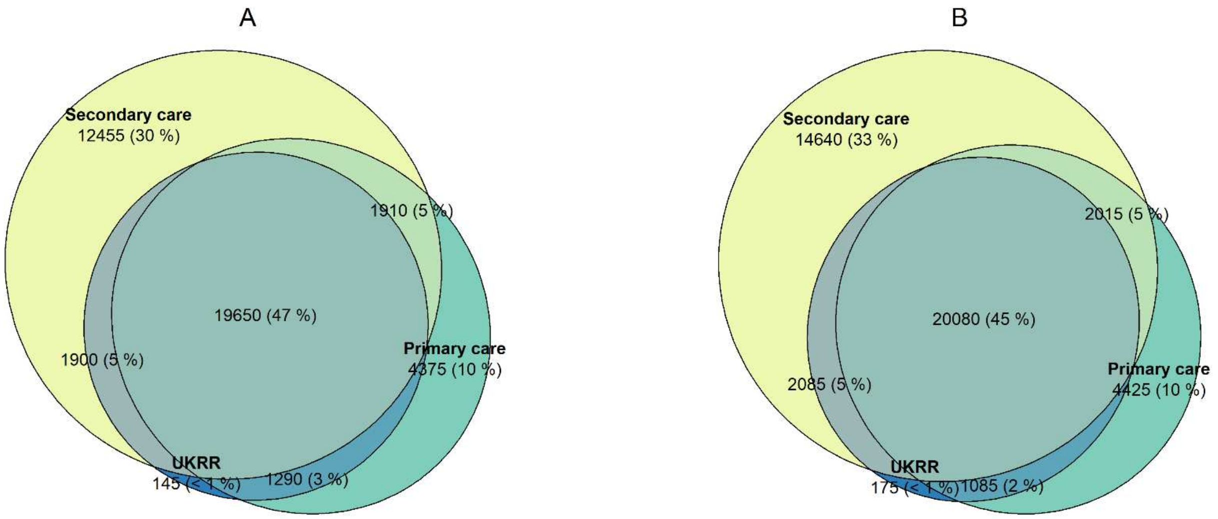

Supplement: Supplementary data [file bmjmed-2023-000807supp001.pdf]
